# Contrasting patterns of gene expression indicate differing pyrethroid resistance mechanisms across the range of the New World malaria vector *Anopheles albimanus*

**DOI:** 10.1371/journal.pone.0210586

**Published:** 2019-01-30

**Authors:** Lucy Mackenzie-Impoinvil, Gareth D. Weedall, Juan C. Lol, Jesús Pinto, Lucrecia Vizcaino, Nicole Dzuris, Jacob Riveron, Norma Padilla, Charles Wondji, Audrey Lenhart

**Affiliations:** 1 Entomology Branch, Division of Parasitic Diseases and Malaria, Center for Global Health, Centers for Disease Control and Prevention, Atlanta, Georgia, United States of America; 2 Vector Biology Department, Liverpool School of Tropical Medicine, Liverpool, United Kingdom; 3 School of Natural Sciences and Psychology, Liverpool John Moores University, Liverpool, United Kingdom; 4 Centro de Estudios en Salud, Universidad del Valle de Guatemala, Guatemala; 5 Instituto Nacional de Salud Lima, Lima, Peru; 6 Centre for Research in Infectious Diseases (CRID), Yaoundé, Cameroon; University of Crete, GREECE

## Abstract

Decades of unmanaged insecticide use and routine exposure to agrochemicals have left many populations of malaria vectors in the Americas resistant to multiple classes of insecticides, including pyrethroids. The molecular basis of pyrethroid resistance is relatively uncharacterised in American malaria vectors, preventing the design of suitable resistance management strategies. Using whole transcriptome sequencing, we characterized the mechanisms of pyrethroid resistance in *Anopheles albimanus* from Peru and Guatemala. *An*. *albimanus* were phenotyped as either deltamethrin or alpha-cypermethrin resistant. RNA from 1) resistant, 2) unexposed, and 3) a susceptible laboratory strain of *An*. *albimanus* was sequenced and analyzed using RNA-Seq. Expression profiles of the three groups were compared based on the current annotation of the *An*. *albimanus* reference genome. Several candidate genes associated with pyrethroid resistance in other malaria vectors were found to be overexpressed in resistant *An*. *albimanus*. In addition, gene ontology terms related to serine-type endopeptidase activity, extracellular activity and chitin metabolic process were also commonly overexpressed in the field caught resistant and unexposed samples from both Peru and Guatemala when compared to the susceptible strain. The cytochrome P450 CYP9K1 was overexpressed 14x in deltamethrin and 8x in alpha-cypermethrin-resistant samples from Peru and 2x in deltamethrin-resistant samples from Guatemala, relative to the susceptible laboratory strain. CYP6P5 was overexpressed 68x in deltamethrin-resistant samples from Peru but not in deltamethrin-resistant samples from Guatemala. When comparing overexpressed genes between deltamethrin-resistant and alpha-cypermethrin-resistant samples from Peru, a single P450 gene, CYP4C26, was overexpressed 9.8x (p<0.05) in alpha-cypermethrin-resistant samples. In Peruvian deltamethrin-resistant samples, the knockdown resistance mutation (*kdr*) variant alleles at position 1014 were rare, with approximately 5% frequency, but in the alpha-cypermethrin-resistant samples, the frequency of these alleles was approximately 15–30%. Functional validation of the candidate genes and the *kdr* mutation as a resistance marker for alpha-cypermethrin will confirm the role of these mechanisms in conferring pyrethroid resistance.

## Introduction

Between 2010 and 2016, malaria cases declined by 22% in the Americas. However, despite these long-term reductions, recent substantial increases in case incidence were detected between 2014 and 2016, with 9 out of 11 countries showing an increase of more than 20% in malaria cases between 2015 and 2016 [1]. Of the 21 malaria-endemic countries in the region, 7 are currently categorized as being in either pre-elimination or elimination stages, yet an estimated 120 million people remain at risk of infection, with 25 million people considered to be at high risk [2]. Vector control interventions using insecticides, such as indoor residual spraying (IRS) and long lasting insecticide-treated nets (LLINs), remain a cornerstone of malaria prevention and control. However, their efficacy is threatened by the development of insecticide resistance in mosquitoes [2].

Pyrethroids are the insecticide class most commonly used for mosquito control. However, their widespread use has driven the evolution of highly resistant vector populations [3–5] and pyrethroid ineffectiveness is increasingly reported in areas where pyrethroid-treated bednets and pyrethroid-based IRS are used for malaria control [6–8]. In some cases, selection for pyrethroid resistance may be driven or exacerbated by the widespread use of pyrethroids for agricultural and domestic pest control [9, 10]. Mosquitoes can become resistant to insecticides by several mechanisms: mutations in insecticide target proteins such as the voltage-gated sodium channel (*kdr*) [11], acetylcholinesterase (AChE) [12] or gamma-aminobutyric acid receptors [13] can lead to target-site insensitivity; increased biodegradation of insecticides can occur due to enhanced detoxification by key metabolic enzymes such as cytochrome P450 monooxygenases, glutathione S-transferases and esterases [14]; thickening of the insect’s cuticle [15] or behavioural avoidance [16] of insecticide treated surfaces can also increase survival. In order to facilitate resistance management, it is crucial to characterise the resistance mechanisms in a region. Markers for target site mutations such as kdr and ace-1 exist but on the other hand, few such markers exist for metabolic resistance which is more complex due to the high diversity of genes potentially involved in insecticide degradation pathways [3, 17]. Metabolic detoxification has also been shown to be driven by a set of enzymes which are often specific to an insecticide. Elevated levels of cytochrome P450 are often observed in pyrethroid-resistant mosquitoes [18–20] while glutathione-s-transferases are a key metabolic mechanism of DDT resistance [21]. As a result, there is an urgent need to disentangle metabolic resistance mechanisms to detect the main genes involved in resistance to certain insecticides and to evaluate the risk of cross-resistance between insecticide classes. This has led to the application of high-throughput techniques such as RNA-Seq to study the transcriptomes of vectors of differing resistance phenotypes, enabling the identification of candidate genes that can be utilized to develop novel markers for resistance surveillance [22].

*Anopheles albimanus* is one of the principal malaria vectors in the Americas. Distributed throughout Central America, South America and the Caribbean Islands where it is an important contributor to malaria transmission in many coastal areas [23]. Resistance to several insecticides has been reported in *An*. *albimanus* [24–29], and resistance patterns often appear to be associated with agricultural insecticide use [30–33], such as where larval habitats coincide with areas of banana and rice cultivation [30]. Malathion resistance reported in the 1960s in Central America was attributed to the proximity of mosquito larval habitats to cotton-growing areas with heavy use of organophosphates to control agricultural pests [34, 35].

Compared to African malaria vectors, little is known about the mechanisms of insecticide resistance in malaria vectors in the Americas, and this is the first study to elucidate resistance mechanisms and detect genes driving pyrethroid resistance in a New World malaria vector. Elucidation of the specific genes and mechanisms conferring resistance in *An*. *albimanus* will allow for a more comprehensive understanding of how resistance develops and could potentially be managed in the field.

## Materials and methods

### Study sites and samples

**Fig 1:**
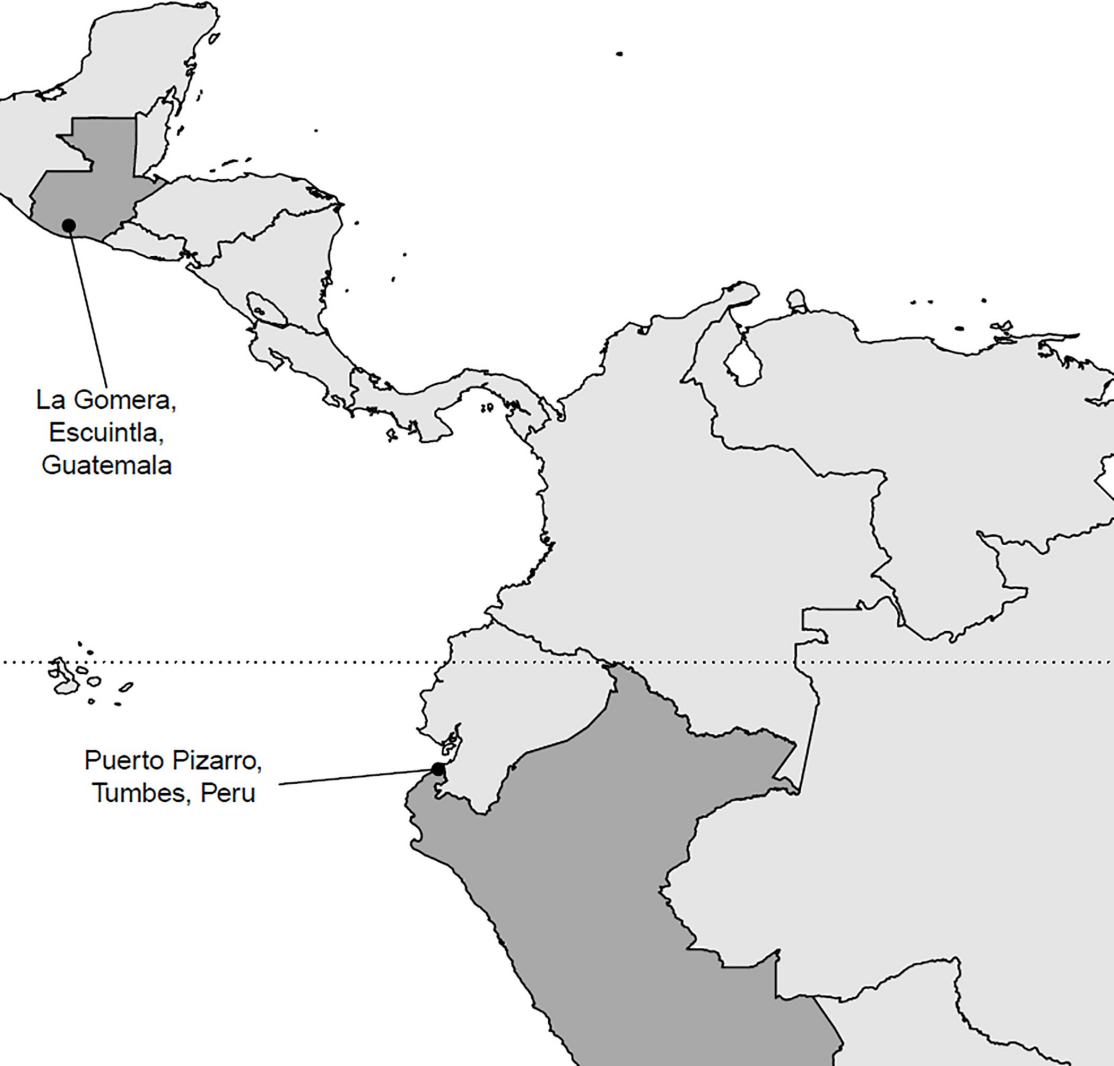
Locations from which mosquitoes were sampled in this study. Guatemala and Peru are shaded in dark grey and sample locations are labelled. The dotted line indicates the equator.

Adult female *An*. *albimanus* were collected in October 2015 from Puerto Pizarro, Tumbes, Peru (3° 30' 10S, 80° 23' 38 W). Female mosquitoes were aspirated from livestock corrals and transported live to the laboratory at the Instituto Nacional de Salud laboratory in Tumbes for morphological identification and bioassay screening. Adult mosquitoes from El Terrero, La Gomera in Escuintla, Guatemala (14°08’ 31” N and 91°05’ 54”W) were collected in November 2015 from livestock corrals which were located near sugarcane plantations and transported to the insectary at the Center for Health Studies of Universidad del Valle de Guatemala in Guatemala City, where they were morphologically identified before bioassay screening. (Fig 1). Attempts to rear offspring of field-collected mosquitoes were unsuccessful, so field-collected adult mosquitoes were used for the bioassays. Mosquito samples from both sites were collected from livestock corrals that were located on privately owned farms. Verbal permission to enter the corrals to collect the mosquitoes was obtained from the farm owners. No protected or endangered species were involved in the field studies.

*An*. *albimanus* mosquitoes from the insecticide susceptible Sanarate laboratory colony, originating from Guatemala, were reared in the insectary at the Centers for Disease Control and Prevention (CDC), Atlanta, Georgia, USA. Mosquitoes were maintained at a constant 27 ± 2°C and 70 ± 10% humidity on a 14:10 hour light:dark cycle and adults were provided 10% sucrose *ad libitum*.

### Insecticide susceptibility testing

Field-collected adult *An*. *albimanus* were tested for resistance to the diagnostic doses of deltamethrin (12.5 μg/bottle) and alpha-cypermethrin (20.5 μg/bottle) using the CDC bottle bioassay [36]. Assays were carried out in four replicates, each containing approximately 25 individuals (range 23–38) per bottle. Mosquitoes alive after 30 minutes exposure to insecticide were considered resistant. These are the two main insecticides used for public health in Peru and Guatemala.

### RNA extraction, RNA-Seq library preparation and sequencing

Three to five day-old adult non-bloodfed female mosquitoes from the Sanarate colony were killed by freezing and stored at -80°C until RNA extraction. For field-collected mosquitoes used in the bottle bioassays, mosquitoes alive at the end of the 30 minute exposure period were considered resistant. Both the resistant mosquitoes and unexposed individuals from each population were briefly chilled on ice and submerged in RNAlater, then shipped to the laboratory at the CDC, Atlanta, USA for molecular analysis. For RNA-Seq analysis, 3 biological replicates with pools of 5 mosquitoes each were prepared according to the following: the susceptible laboratory colony Sanarate (San); field-collected mosquitoes from Guatemala not exposed to insecticide (GTM-unx) and alive after exposure to deltamethrin (GTM-delta); field-collected mosquitoes from Peru not exposed to insecticide (PER-unx), alive after exposure to deltamethrin (PER-delta) and alive after exposure to alpha-cypermethrin (PER-acyp). The number of mosquitoes from La Gomera, Guatemala alive after exposure to alpha-cypermethrin was insufficient for RNA extraction hence RNA-Seq analysis was not done for this group. RNA was extracted using the Applied Biosystems Arcturus PicoPure RNA isolation kit (Arcturus, Applied Biosystems, USA) according to the manufacturer’s instructions. RNA concentration and integrity were assessed using the Qubit 3.0 Fluorometer (Life Technologies, Carlsbad, CA, USA) and Agilent 2100 Bioanlyzer (Agilent Technologies, Palo Alto, CA, USA), respectively. RNA was DNase treated using Baseline-ZERO DNase (Epicentre, Illumina) and ribosomal RNA depleted using Ribo-Zero rRNA removal kit (Human/Mouse/Rat) (Epicentre, Illumina). Library preparation was carried out using the ScriptSeq v2 RNA-Seq Library Preparation Kit (Epicentre, Illumina) according to the manufacturer’s instructions. Each library was barcoded and equal amounts of each library pooled and sequenced (2x125bp paired-end) on an Illumina HiSeq 2500 sequencer, using v2 chemistry. Sequencing was done at the Biotechnology Core Facility at CDC, Atlanta, USA.

### RNA-Seq data analysis

Sequencing reads were trimmed to remove Illumina adapter sequence (a match of at least 3 bp from the 3’ end of the read) using cutadapt v1.2.1[37] and to remove low quality sequences using sickle v1.200 [38] with a minimum window quality score of 20. Read pairs where one or both reads were shorter than 25 bp after trimming were removed. Trimmed reads were aligned to the *An*. *albimanus* STECLA reference genome assembly AalbS2 [23] using ‘subjunc’, part of the subread aligner package, version 1.5.0.p [39] with default parameters. Alignments were filtered to remove reads with low mapping quality (<10). Descriptive statistics for the read libraries and sequence alignments are shown in S1 Table. Filtered alignments comprised between 46% and 80% of reads in the libraries.

Aligned read pairs were assigned to genes to quantify the levels of gene expression and these data were used for differential gene expression analyses to compare putatively resistant mosquitoes to (i) unexposed mosquitoes from the same location and (ii) the fully insecticide susceptible laboratory colony. The alignments were used to quantify gene expression of the gene set AalbS2.1. Tag counting (a ‘tag’ being a read pair or single, unpaired read) was done using ‘featurecounts’, part of the subread aligner package, version 1.5.0.p [39, 40] Aligned reads/pairs that overlapped coding sequence (CDS) features by at least 1 bp in the sense orientation were counted. Tabulated tag counts were used as input for differential expression analysis using edgeR [41]. To remove the effect of noise and very lowly expressed genes, only genes where at least one sample had a tag count of 50 or more were analysed. For comparisons of field-collected and laboratory colony mosquitoes, a critical value of 1% and absolute fold-change of 2x were used to define differentially expressed genes. For comparisons among field-collected mosquitoes, values of 5% and 1.5x were used.

Candidate resistance genes were identified based on the assumption that they are significantly differentially expressed between resistant and susceptible mosquitoes. Comparisons were done between resistant field mosquitoes and unexposed field mosquitoes (PER-delta vs PER-unx, PER-acyp vs PER-unx and GTM-delta vs GTM-unx), between resistant field mosquitoes and laboratory susceptible mosquitoes (PER-delta vs San, PER-acyp vs San and GTM-delta vs San), between mosquitoes resistant to different insecticides within the same geographical location (PER-delta vs PER-alpha), and between mosquitoes from different geographical location resistant to the same insecticides (PER-delta vs GTM-delta).

Gene ontology enrichment analysis was carried out on differentially expressed gene sets using blast2go [42]. Fisher’s exact test was used to identify gene ontologies significantly enriched in over- and under-expressed gene sets relative to the rest of the genome.

### Functional annotation improvement of gene set AalbS2.1

Our analysis used the *An*. *albimanus* STECLA reference genome assembly AalbS2 and annotation gene set AalbS2.1 [43], downloaded from VectorBase [44]. The annotation gene set AalbS2.1 includes 12,232 protein coding gene annotations (corresponding to 12,110 genes, with 122 additional isoforms). However, functional descriptions exist for only 780 of these. To aid interpretation of results, all AalbS2.1 predicted proteins were used for similarity-based functional annotation assignment using blast2go [42]. BLASTp searches of the non-redundant protein database (nr) and InterProScan searches of the InterPro protein signature databases were carried out and the results used to assign descriptions and gene ontologies to the *An*. *albimanus* proteins. This analysis assigned putative descriptions to 10,948 protein coding genes and gene ontology descriptions to 9,117. All annotations are shown in S2 Table.

### Identifying target site mutations using RNA-Seq datasets

To identify target site mutations in the different mosquito populations, RNA-Seq alignments from pools of mosquitoes from the Sanarate laboratory colony and field-collected mosquitoes from Guatemala (GTM-unx and GTM-delta) and Peru (PER-unx, PER-delta, PER-acyp), were inspected at the relevant codon positions. SNPs were only counted from reads that completely spanned the codon. The primary target of the analysis was the *kdr* mutation in the *para* voltage gated sodium channel (VGSC) gene, conferring resistance to pyrethroids and DDT. Two additional target site mutations were also analysed: one in the Acetylcholinesterase-1 (ACE-1) gene, associated with carbamate and organophosphate resistance, and one in the GABA gated chloride channel A (GABA-a) gene, associated with resistance to the organochlorine dieldrin. The voltage gated sodium channel gene (AALB007478) codon ‘1014’, containing *kdr* (‘knockdown resistance’) mutations is at positions 3R:32,695,670–72 and the codon is TTG (Leucine, susceptible) in STECLA. The Acetylcholinesterase-1 gene (AALB002313) codon ‘119’, containing ACE-1 mutations, the codon is GGC (Glycine, susceptible) in STECLA. The GABA gated chloride channel (AALB015766) codon ‘296’, containing known *Rdl* (‘resistance to dieldrin’) mutations in other *Anopheles* species, the codon is TGC (Alanine, susceptible) in STECLA.

### Measurement of gene expression by quantitative PCR

Candidate genes that were significantly differentially expressed in the RNA-Seq analysis were validated using quantitative PCR (qPCR). One microgram of RNA from 3 replicates of samples resistant to alpha-cypermethrin from Peru were used to synthesize cDNA using the High-Capacity cDNA reverse transcription kit (Applied Biosystems) with oligo-dT20 (NEB), according to the manufacturer’s instructions. Only samples resistant to alpha-cypermethrin from Peru were used for qPCR validation, as there was insufficient material remaining from deltamethrin-resistant samples from Peru or Guatemala. The primers used are listed in S3 Table. Standard curves of Ct values for each gene were generated using a serial dilution of cDNA, allowing assessment of PCR efficiency. The qPCR amplification was carried out on a QuantStudio 6 Flex Real-Time PCR system (Applied Biosystems) using PowerUp SYBR Green Master Mix (Applied Biosystems). cDNA from each sample was used as a template in a three-step program: 50°C for 2 minutes denaturation at 95°C for 10 minutes, followed by 40 cycles of 15 seconds at 95°C, 1 minute at 60°C and a last step of 15 seconds at 95°C, 1 minute at 60°C, and 15 seconds at 95°C.

The relative expression level and Fold Change (FC) of each target gene from resistant field samples relative to the susceptible lab strain were calculated using the 2^−ΔΔCT^ method [45] incorporating the PCR efficiency. Housekeeping genes ribosomal protein S17 (RPS17; AALB004745) and Actin (AALB015449) were used for normalisation. A two-sample t-test was used to assess the statistical significance of the results between samples.

### Identifying Cytochrome Oxidase I (COI) haplogroups of field-collected *An*. *albimanus* from Guatemala and Peru

To identify genetic populations to which the field-collected mosquitoes belonged, we identified the genotypes of the COI and compared them to published data for this gene. A 1,510 bp sequence, KC354825.1, was chosen as a reference sequence and RNA-Seq reads were aligned to it using subjunc [39]. Alignment statistics are shown in S6 Table. Alignments were viewed using the Integrative Genomics Viewer, IGV [46], which was used to obtain consensus sequences for each sample. These were added to a set of 112 sequences derived from mosquitoes from Pacific and Caribbean Colombia [47] and 27 sequences derived from mosquitoes from throughout Panama [48]. All sequences were aligned using Muscle [49] within the SeaView alignment editor [50]. The alignment was edited to remove poorly aligning sequences and trimmed to regions common to all sequences. This resulted in an alignment of 776 bp. Sequence similarity was visualised using a multi-dimensional scaling plot, generated in *R*.

## Results

### Resistance profiles of *An*. *albimanus* from Guatemala and Peru

**Fig 2:**
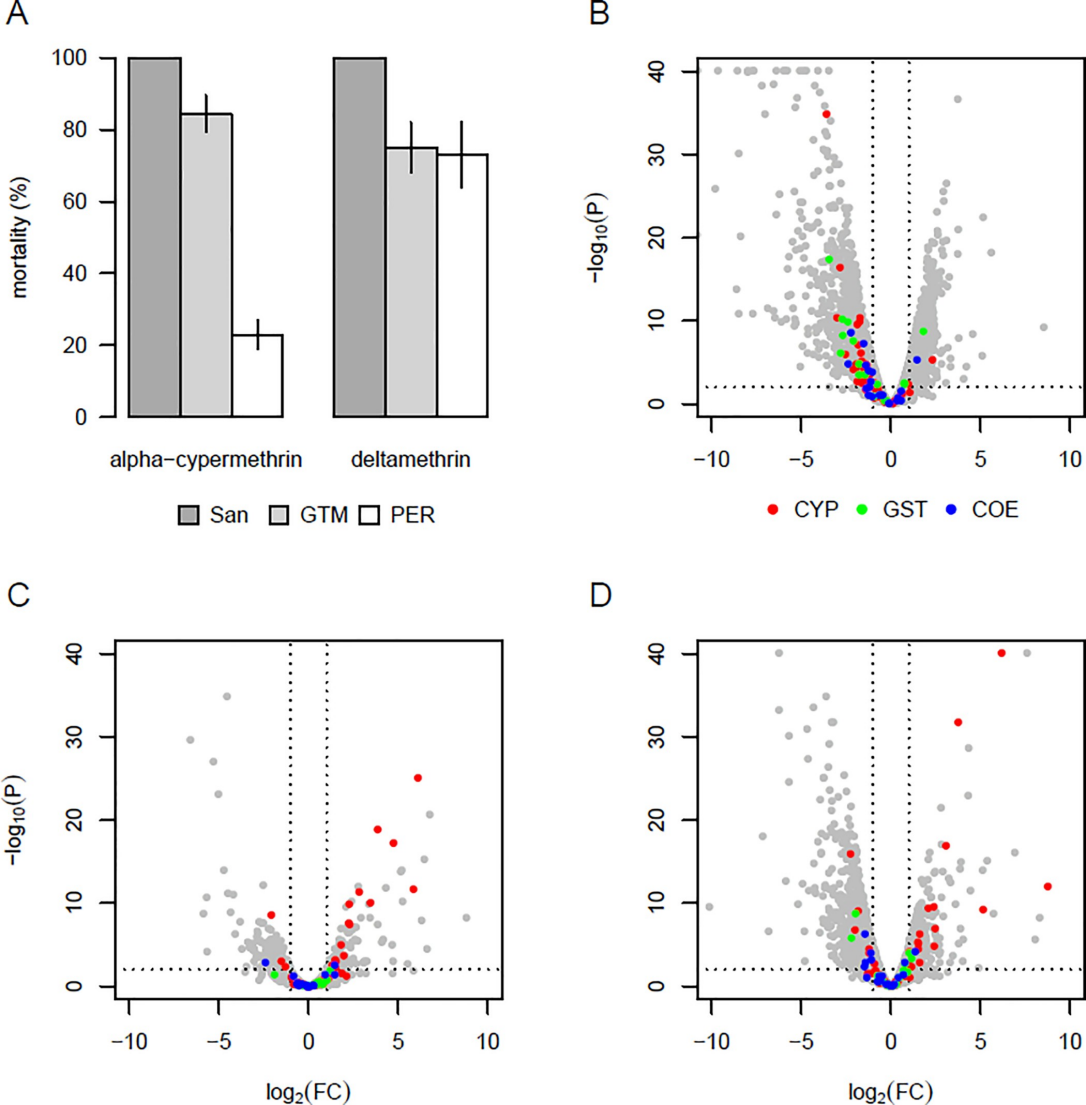
Pyrethroid resistance and gene expression profiles in *An*. *albimanus*. (A) Percent mortality in bottle bioassays for the susceptible Sanarate strain (dark grey) and field populations from Guatemala (light grey) and Peru (white). Bars show the means of 4 replicate assays, with error bars indicating +/-1 standard error of the mean. Remaining panels show volcano plots of gene expression for the comparisons: (B) Guatemala deltamethrin resistant *vs*. Sanarate; (C) Peru deltamethrin resistant *vs*. Sanarate; (D) Peru alpha-cypermethrin resistant *vs*. Sanarate. Red, green and blue points on the volcano plots indicate three gene families with major roles in metabolic resistance to insecticides: cytochrome P450 monooxygenases (CYP, red), glutathione S-transferases (GST, green) and carboxylesterases (COE, blue). In each plot, genes overexpressed in the population are >0 on the x-axis. Vertical dotted lines indicate 2-fold expression differences and the horizontal dotted line indicates a p-value of 0.01.

A total of 123 mosquitoes from Tumbes, Peru were phenotyped as alpha-cypermethrin resistant or susceptible and 121 phenotyped as deltamethrin resistant or susceptible. After 30 minutes insecticide exposure, mosquitoes from Tumbes, Peru exhibited a mortality of 23.1% (SEM = 4.05) for alpha-cypermethrin (n = 123) and 73.1% (SEM = 9.11) for deltamethrin (n = 121). Mosquitoes from Escuintla, Guatemala exhibited a mortality of 84.5% (SEM = 5.07) for alpha-cypermethrin (n = 97) and 75.0% (SEM = 7.00) for deltamethrin (n = 100). A hundred and two and a hundred and six mosquitoes from the susceptible Sanarate colony were phenotyped for alpha-cypermethrin and deltamethrin resistance respectively and showed 100% mortality as expected (Fig 2A).

### Differential gene expression analyses

Results of differential gene expression analyses to compare the transcriptomic profiles of the different mosquito populations and identify genes associated with insecticide resistance are summarised in Table 1. Full results for all analyses are presented in S4 Table, and results of gene ontology enrichment analyses for sets of differentially expressed genes are shown in S5 Table.

**Table 1:** Summary of results of differential gene expression analyses. DE = differentially expressed, FC = Fold change and adjP = P-value adjusted for multiple testing by the method of Benjamini and Hochberg 1995 [51].

Condition-1	Condition-2	Genes	DE genes (adjP<0.05)	DE genes (adjP<0.01)	Condition-2 down	Condition-2 up	Condition-2 down	Condition-2 up
		tested			(|FC|>2 & adjP<0.01)	(|FC|>2 & adjP<0.01)	(|FC|>1.5 & adjP<0.05)	(|FC|>1.5 & adjP<0.05)
San	GTM-delta	8,720	4,743	3,726	1,852	978	2,494	2,010
San	GTM-unx	8,860	6,122	5,336	2,414	1,421	3,021	2,619
GTM-unx	GTM-delta	8,502	353	105	3	76	39	276
San	PER-delta	8,785	565	302	167	135	298	267
San	PER-unx	8,840	3,294	2,105	1,196	355	1,891	1,270
San	PER-acyp	8,779	4,088	2,935	1,376	556	2,138	1,665
PER-unx	PER-delta	8,656	7	5	5	0	5	2
PER-unx	PER-acyp	8,577	6	5	3	2	4	2
PER-delta	PER-acyp	8,585	16	9	5	4	8	8
GTM-delta	PER-delta	8,630	2,859	1,893	564	1,157	1,198	1,661

#### Differential gene expression associated with resistance

Detection of genes potentially associated with deltamethrin and alpha-cypermethrin resistance followed a gradual analysis with the hypothesis that the best candidate genes should be significantly differentially expressed (p<0.05 and fold change (FC) >2) in most of the comparisons between resistant, unexposed control and susceptible mosquitoes. A three pairwise comparison was conducted for each insecticide: resistant vs susceptible (R-S), resistant vs unexposed control (R-C) and unexposed control vs susceptible (C-S). We chose this approach in order to account for geographical background differences in the resistant vs susceptible (R-S) comparison, while the resistant vs unexposed control (R-C) comparison was used to account for genes overexpressed due to induction and the unexposed control vs susceptible (C-S) comparison was used to account for genes that are constitutively overexpressed. With this approach, the genes that are consistently over-expressed in different comparisons provide the best evidence of involvement in insecticide resistance.

**Fig 3:**
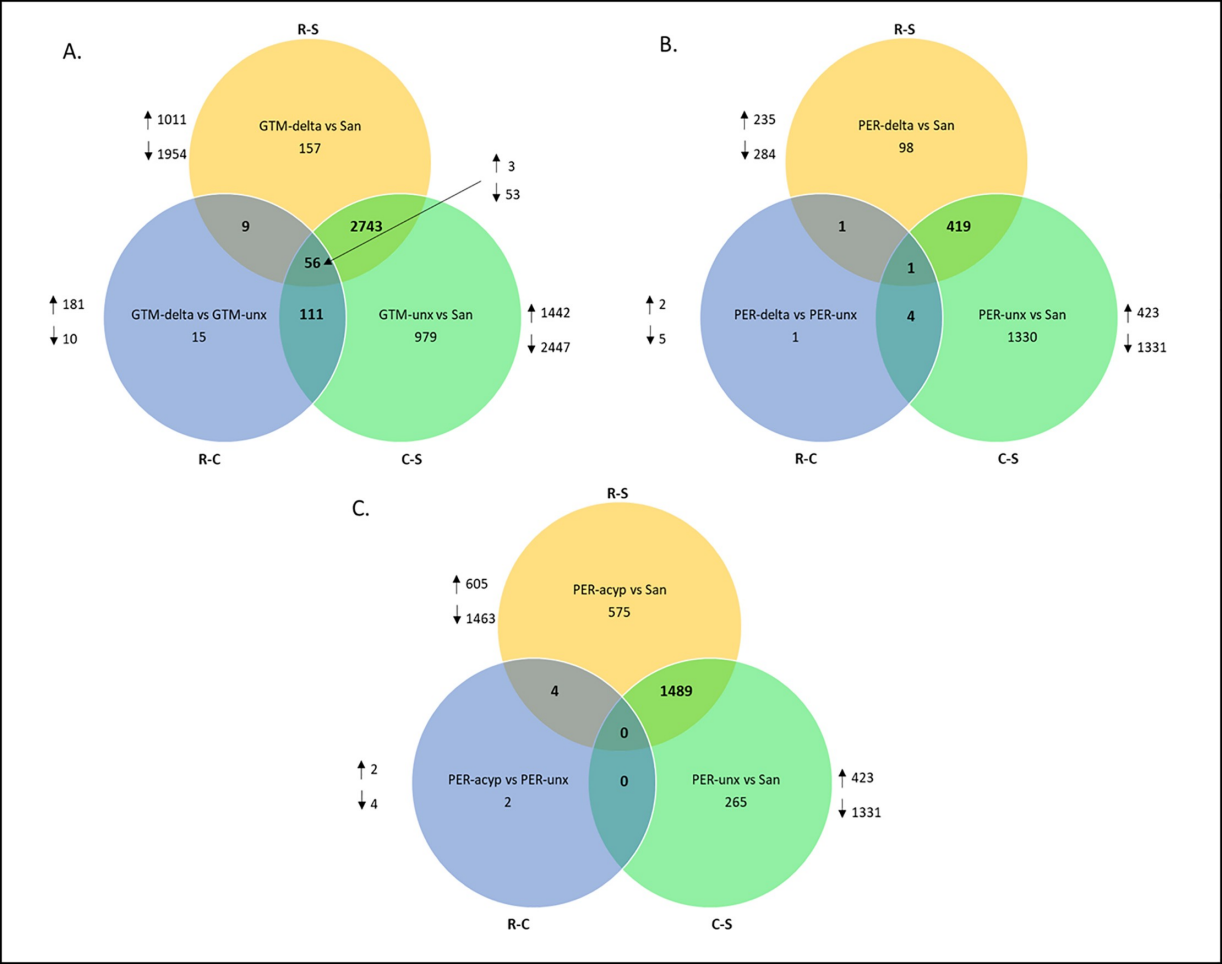
Venn diagrams summarizing the numbers of differentially expressed (DE) genes between resistant (R), unexposed (C) and susceptible (S) samples with a transcription ratio ≥ 2 fold change in either direction and a corrected p value < 0.05. A.) DE genes in the Guatemala deltamethrin resistant samples; B.) DE genes in the Peru deltamethrin resistant samples; C.) DE genes in the Peru alpha-cypermethrin samples.

**Differential gene expression associated with deltamethrin resistance**. Comparing Guatemalan mosquitoes surviving deltamethrin exposure to the Sanarate susceptible strain showed highly different transcriptomic profiles. The number of genes significantly and differentially expressed (DE) for R-S 2965 (1011 up-regulated and 1954 down-regulated) and C-S 3889 (1442 up-regulated and 2447 down-regulated) was quite high The R-C group had 191 differentially expressed genes (181 up-regulated and 10 down-regulated) (Fig 3A).

In comparing genes commonly differentially expressed in (R-C)/(R-S)/(C-S), among the 56 genes commonly overexpressed in all three comparisons, 3 were upregulated while 53 were down regulated. Two of the overexpressed genes belong to the proteolysis activity GO term (FC = 35.31 and 3.43) and one to the extracellular space GO term (FC = 2.66). Overexpression of genes from detoxification gene families associated with metabolic resistance (cytochrome P450 monooxygenases, glutathione S-transferases, and carboxylesterases) were not generally apparent. Two glutathione-S-transferases GSTs1 (AALB007583, FC = 0.188) and GSTU1 (AALB006654, FC = 0.368) were down regulated in the R-S comparison and even lower in the C-S comparison (FC = 0.085 and FC = 0.18). Two cytochrome P450 genes also down regulated were CYP047 (AALB015530, FC = 0.383) and cytochrome b-561 (AALB005960, FC = 0.479) in R-S, and in C-S (FC = 0.194 and FC = 0.203). One putative glucuronosyltransferase was also down regulated (AALB003189, FC = 0.45 in R-S and FC = 0.18 in C-S).

**Fig 4:**
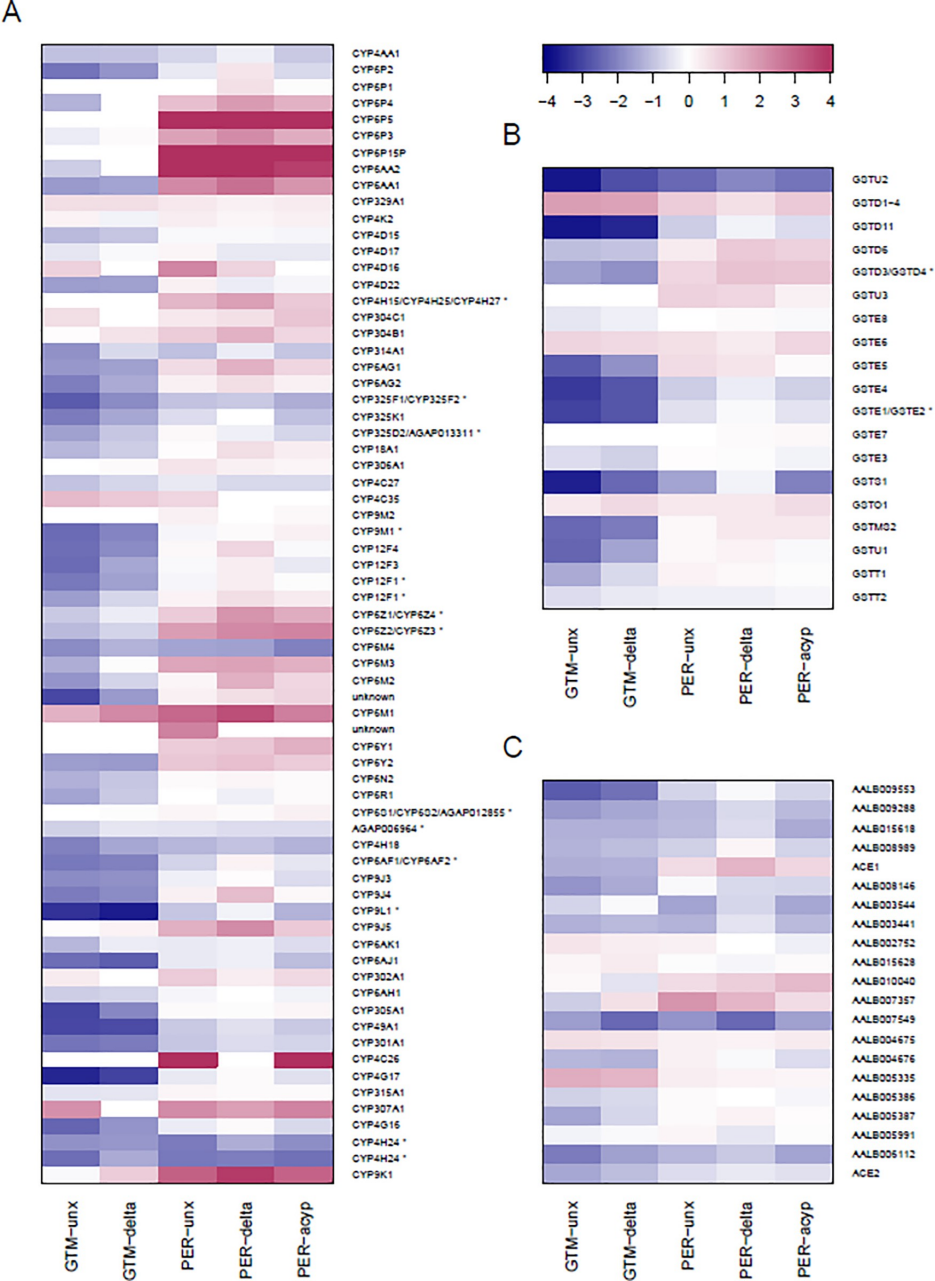
Heatmaps summarizing expression of detoxification genes, showing log_2_ fold-change values relative to the susceptible strain Sanarate on a blue-red scale (red = overexpressed). Gene families are: (A) cytochrome P450 monooxygenases; (B) glutathione S-transferases; (C) carboxylesterases. Gene names are based on orthology to *An*. *gambiae* (an asterisk denotes ambiguous orthology). Genes on chromosome arms 2R, 2L, 3R, 3L and X are ordered from top to bottom. San = Sanarate; GTM = Guatemala; PER = Peru; unx = unexposed; delta = deltamethrin-resistant; acyp = alpha-cypermethrin-resistant.

A total of 2743 genes were differentially expressed commonly in the R-S and C-S groups (Fig 3A). Among the top 10 genes, were four genes representing the DNA and RNA binding GO terms in R-S (AALB007073, FC = 47.13, AALB015691, FC = 23.81, AALB008478, FC = 13.73 and AALB001046, FC = 13.55 and in C-S (AALB007073, FC = 30.974, AALB015691, FC = 22.721, AALB008478, FC = 10.68 and AALB001046, FC = 17.37).One belonged to the serine-type endopeptidase activity (AALB007819, FC = 34.05 in R-S; FC 33.707 in C-S) and one to the chromosome passenger complex group (AALB010585, FC = 13.20 in both R-S and C-S). The remaining four genes were not annotated. Among the remaining genes, FigFigseven glutathione-S-transferase (GST) genes GSTd1, GSTd3, GSTe5 GSTe2 GSTe4 GSTu2 and GSTD11 were down regulated and GSTd1 was upregulated (AALB015606, FC = 3.53 in R-S and FC = 3.75 in C-S). Twenty-eight cytochrome P450s were DE with only two that were upregulated in R-S, CYP6M1 (AALB015585, FC = 5.00 in R-S and FC = 2.80 in C-S) and CYP4C35 (AALB001020, FC = 2.08 in R-S and FC = 2.53 in C-S) (Fig 2B, Fig 4). Twenty-six cuticular proteins were DE with only one overexpressed belonging to the CPLCG family (AALB000605, FC = 2.80 in R-S and FC = 3.80 in C-S). Five putative UDP- glucuronosyltransferase were downregulated in R-S and C-S (Table 2).

**Table 2:** Differentially expressed detoxification genes common in the comparisons of resistant vs. susceptible (R-S) and control vs. susceptible (C-S) groups, with a corrected p value < 0.05. Gene description is based on orthology to *An*. *gambiae*.

Gene ID	Gene description	GTM-delta R-S fold change	GTM-unx C-S fold change	PER-delta R-S fold change	PER-acyp R-S fold change	PER-unx C-S fold change
AALB015620	CYP6P5			68.60	72.57	63.34
AALB015617	CYP6P15P			58.10	35.47	34.58
AALB015588	CYP6AA2			26.75	13.48	22.20
AALB003283	CYP9K1			14.43	8.36	8.71
AALB015585	CYP6M1	5.00	2.79	10.93	5.58	7.74
AALB015589	CYP6AA1	0.37	0.33	7.08	4.29	4.91
AALB015455	CYP6Z2/CYP6Z3			4.92	5.23	3.69
AALB015619	CYP6P3			4.83	3.06	3.43
AALB010082	CYP9J5			4.74		2.89
AALB015621	CYP6P4			3.92	2.84	2.37
AALB006365	CYP307A1			3.65		4.82
AALB015703	CYP4H15/CYP4H25/CYP4H27			3.55		2.62
AALB015735	CYP6M3			3.50	2.89	3.38
AALB015574	CYP6Y2	0.33	0.34	2.36		2.11
AALB015507	CYP4H24	0.32	0.30	0.41	0.28	0.23
AALB015737	CYP6M4	0.44	0.28	0.35	0.25	0.37
AALB015506	CYP4H24	0.40	0.20	0.24	0.21	0.23
AALB015526	CYP4C26				429.96	384.10
AALB001852	Cytochrome P450				3.37	3.67
AALB001475	CYP4H18	0.38	0.25		0.43	0.46
AALB010702	Cytochrome P450				0.41	0.41
AALB001020	CYP4C35	2.08	2.53			
AALB015515	CYP6AG2	0.39	0.24			
AALB008236	CYP325K1	0.38	0.24			
AALB015514	CYP6AG1	0.36	0.32			
AALB015535	CYP4D22	0.35	0.34			
AALB015528	CYP12F1	0.35	0.23			
AALB015584	cytochrome_P450	0.32	0.13			
AALB015623	CYP6P2	0.31	0.22			
AALB007673	CYP4G16	0.31	0.18			
AALB015652	CYP9J3	0.30	0.27			
AALB015531	CYP12F4	0.28	0.21			
AALB015651	CYP9J4	0.28	0.24			
AALB015552	CYP305A1	0.26	0.13			
AALB015509	CYP9M1	0.26	0.20			
AALB006158	CYP301A1	0.23	0.22			
AALB007373	CYP6AJ1	0.17	0.20			
AALB006097	CYP49A1	0.14	0.13			
AALB006842	CYP4G17	0.12	0.09			
AALB015653	CYP9L1	0.08	0.10			
AALB008278	CYP325F1/AgCYP325F2	027	0.17			
AALB009920	CYP6AF1/AgCYP6AF2	0.25	0.22			
AALB015585	CYP6M1	5.00	2.79			
AALB001020	CYP4C35	2.08	2.53			
AALB000333	Putative glucuronosyltransferase			7.64	7.06	7.05
AALB005262	Putative glucuronosyltransferase			3.63	2.27	2.83
AALB010106	Putative glucuronosyltransferase			2.65	2.79	2.77
AALB004669	Putative glucuronosyltransferase			4.62	2.1	2.71
AALB015521	Putative glucuronosyltransferase			3.53	2.83	2.48
AALB006205	Putative glucuronosyltransferase			2.76	2.19	2.16
AALB006205	Putative glucuronosyltransferase	0.40	0.32			
AALB015524	Putative glucuronosyltransferase	0.40	0.26			
AALB001015	Putative glucuronosyltransferase	0.39	0.29			
AALB006116	Putative glucuronosyltransferase	0.34	0.16			
AALB015522	Putative glucuronosyltransferase	0.28	0.16			
AALB015606	glutathione S-transferase (GSTd1)	3.54	3.76		2.06	2.01
AALB015683	glutathione S-transferase (GSTd3)	0.29	0.36			
AALB015734	glutathione S-transferase (GSTe5)	0.29	0.16			
AALB015731	glutathione S-transferase (GSTe2)	0.16	0.12			
AALB015733	glutathione S-transferase (GSTe4)	0.15	0.11			
AALB015697	glutathione S-transferase (GSTu2	0.14	0.08	0.27	0.22	0.19
AALB015675	glutathione S-transferase (GSTd11)	0.09	0.08			
AALB007583	glutathione S-transferase (GSTs1)			0.25	0.25	0.25

Comparing Peruvian mosquitoes surviving deltamethrin exposure to the susceptible strain showed a smaller number of DE genes: The number of genes significantly differentially expressed (DE) for the R-S comparison was 519 (235 up- regulated and 284 down-regulated) and 1754 for the C-S comparison (423 up-regulated and 1331 down-regulated). The R-C group had only 7 (two up-regulated and five down-regulated) (Fig 3B).

In comparing genes commonly differentially expressed in (R-C)/(R-S)/(C-S), there was only one common gene belonging to the calcium ion binding GO term in this comparison which was down regulated (AALB000398 FC = 0.043 in R-S and FC = 0.214 in C-S and FC = 0.050 in R-C).

There were 419 genes commonly differentially expressed between R-S and C-S, and possibly related to resistance to deltamethrin in Peru. Of the top 10 genes, the top most upregulated gene (AALB010850, FC 107.634 in R-S and 117.539 in C-S) was associated with chitin metabolic process in GO terms. Two genes were associated with serine-type endopeptidase activity, three with the oxidation-reduction process, one with extracellular activity, one with metal ion binding and two were not annotated. Among the top 10 genes associated with insecticide detoxification three genes that were relatively highly overexpressed were CYP6P5 (AALB015620, FC = 68.593 in R-S and 63.338 in C-S), CYP6P15P (AALB015617, FC = 58.081 in R-S and 34.535 in C-S) and CYP6AA2 (AALB015588, FC = 26.722in R-S and 22.192 in C-S). Other genes include one glutathione-S-transferase GSTu2 (AALB015697 R-S FC = 0.26 and C-S FC = 0.18), that was down regulated.

Despite the smaller number of DE genes overall from Peru, a number of genes associated with insecticide resistance were highly overexpressed (Fig 2C, Fig 4). Seventeen cytochrome P450 genes were DE, with 14 upregulated and 3 downregulated in both R-S and C-S. All genes had a fold change that was slightly higher in the R-S group than in the C-S group. The most overexpressed P450 was CYP6P5 (AALB015620, FC 68.60). Six putative UDP-glucuronosyltransferases were overexpressed, with the highest showing a fold change of 7.64 (AALB000333) (Table 2). Other genes included twelve cuticular proteins that were all down regulated in both the R-S and C-S comparisons, one carboxylesterase (AALB007549, R-S FC = 0.18 and C-S FC = 0.31) that was downregulated and one glutathione-S-transferase GSTu2 (AALB015697 FC = 0.27) that was down regulated in R-S and in C-S FC = 0.19.

**Fig 5:**
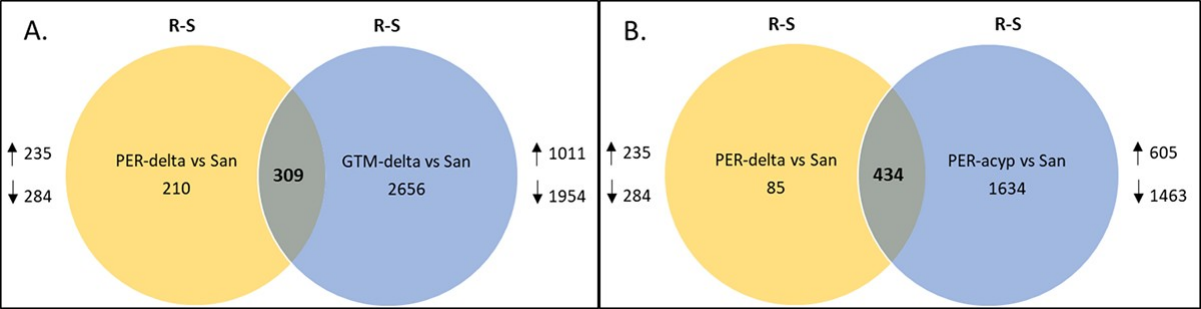
The Venn diagrams summarizing differentially expressed genes with a transcription ratio ≥ 2 fold change in either direction and a corrected p value < 0.05 between A.) Deltamethrin resistant samples from Peru and Guatemala; B.) Deltamethrin and alpha-cypermethrin resistant samples from Peru.

There were 309 genes commonly differentially expressed when a direct comparison of deltamethrin resistant *An*. *albimanus* from Peru and Guatemala was done (Fig 5A). Ten differentially expressed genes belonged to the following GO-terms: two to the chitin metabolic process, four to the serine-type endopeptidase activity, one to oxidation-reductions process and one to transferase activity; two of the genes were not annotated. Genes related to insecticide detoxification, were cytochrome P450 CYP026 (AALB015585, FC 10.93 in Per-delta and 5.00 in GTM-delta) and a UDP-glucuronosyltransferase (AALB000333, FC 7.64 in PER-delta and 2.13 in GTM-delta).

Other genes related to insecticide detoxification among the 309 genes were 12 cuticular genes that were all down regulated in both PER-delta and GTM-delta. More so in the GTM-delta samples. One putative carboxylesterase AALB007549 was down regulated with FC 0.188 in PER-delta and at FC 0.191 in GTM-delta. Two putative UDP-glucuronosyltransferases ((AALB000333, FC 7.64 in PER-delta and FC 2.13 in GTM-delta), (AALB004537, FC 3.61 in PER-delta and FC 3.03 in GTM-delta)) were both overexpressed in the two groups. Unlike the comparisons mentioned above, there were only eight cytochrome P450 shared between the Peruvian deltamethrin resistant mosquitoes and Guatemalan deltamethrin resistant mosquitoes. Of these, only CYP026 (AALB015585, FC 10.93 in PER-delta and FC 5.00 in GTM-delta) was commonly upregulated in both groups. Three cytochrome P450s, CYP030 (AALB015737), CYP043 (AALB015506) and CYP042 (AALB015507) were all down regulated in both groups. The remaining four CYP069 (AALB015589), CYP066 (AALB015514), CYP023 (AALB015574) and CYP056 (AALB015651) were all overexpressed in PER-delta and not in GTM-delta. The differing transcriptomic profiles seen between Guatemala and Peru were also reflected in the patterns of gene ontology enrichment in DE genes (S5 Table). In Peruvian deltamethrin resistant mosquitoes, a small number of ontologies were enriched in genes overexpressed relative to susceptible mosquitoes. Many of these were ontologies associated with cytochrome P450 monooxygenases, such as “iron ion binding” (GO:0005506), “heme binding” (GO:0020037), “oxidoreductase activity, acting on paired donors, with incorporation or reduction of molecular oxygen” (GO:0016705) and “oxidation-reduction process” (GO:0055114). Another, “transferase activity, transferring hexosyl groups” (GO:0016758) may reflect the overexpression of UGTs and several ontologies describing serine-type peptidases activity (GO:0004252, GO:0008236, GO:0017171, GO:0004175) could be associated with the role of peptidases in mitigating stress caused by pesticide exposure [52]. These ontologies also appeared to be enriched in the Peruvian deltamethrin-resistant mosquito samples relative to the Guatemalan deltamethrin-resistant mosquito samples. By contrast, the (larger) gene set overexpressed in Guatemalan deltamethrin resistant mosquitoes relative to susceptible mosquitoes was significantly enriched for a large number of ontologies, many of which were associated with DNA replication and cell cycle processes (S5 Table). This could indicate that the major differences between the mosquito samples compared are differences in the level of active growth and cell proliferation, possibly reflecting a systematic difference in age and/or physiological status of the samples (which could not be controlled for in the field-collected mosquitoes).

**Differential gene expression associated with alpha-cypermethrin resistance.** For the Peruvian mosquitoes surviving alpha-cypermethrin exposure, the number of genes significantly differentially expressed between resistant and susceptible was 2068 (605 upregulated and 1463 downregulated), six (2 upregulated and 4 down-regulated) between resistant and control and 1754 (423 up-regulated and 1331 down-regulated) between control and susceptible (Fig 3C).

There were no DE genes shared between R-S, R-C and C-S groups. However, there were 1489 DE genes common between R-S and C-S. Of the top 10 genes, the top most upregulated gene AALB000941, FC = 2697.544, is not currently annotated but falls under a serine-type endopeptidase inhibitor GO term, followed by a very highly upregulated cytochrome P450 CYP4C26 (AALB015526, FC = 429.960) that only appeared in the alpha-cypermethrin resistant samples. This extreme value is likely due to the gene not being expressed at detectable levels in the susceptible strain, rather than high absolute levels of the AALB015526 transcript in PER-acyp.

Two other detoxification genes among the top 10 were CYP6P5 (AALB015620, FC = 72.567) and CYP6P15P (AALB015617, FC = 35. 469). The other six genes fall in the GO terms serine-type endopeptidases, one to extracellular region, one to sensory perception of taste and one to metal ion binding.

Other genes related to insecticide detoxification were, three glutathione S-transferases one-upregulated (AALB015606 GSTd1 FC = 2.06) and two down-regulated (AALB007583 GSTs1 FC = 0.25 and AALB015697 GSTu2 FC = 0.22) a carboxylesterase that was down- regulated (AALB006112, FC = 0.366), and two down regulated cuticular genes belonging to the cuticular protein RR-1 family. Similar to the Peruvian deltamethrin resistant samples, seventeen cytochrome P450 genes were DE with 12 upregulated and 5 downregulated. Similarly, six putative UDP-glucuronosyltransferases were also overexpressed with the highest showing a fold change of 7.06 (AALB000333) (Table 2, Fig 4).

When alpha-cypermethrin-resistant and deltamethrin-resistant mosquitoes from Peru were compared there were 434 DE genes shared (Fig 5B). Of the top 10 shared genes, three were cytochrome P450s CYP6P5 (AALB015620, FC = 68.601 in PER-delta, FC = 72.567 in PER-acyp, CYP6P15P (AALB015617, FC = 58.101 in PER-delta, FC = 35.469 in PER-acyp) and CYP6AA2 (AALB015588, FC = 26.754 in PER-delta, FC = 13.483 in PER-acyp). The remaining genes fell under the following GO terms: chitin binding (AALB010850, FC = 108.010 in PER-delta; FC = 193.017 in PER-acyp), serine-type endopeptidase (AALB007819, FC = 78.629 in PER-delta; FC = 53.236 in PER-acyp), metal ion binding (AALB007464, FC = 37.896 in PER-delta, FC = 34.647 in PER-acyp), extracellular region (AALB006948, FC = 35.436 in PER-delta; FC = 19.780 in PER-acyp) and acyl-CoA metabolic process (AALB010577, FC = 26.479 in PER-delta and FC = 16.082 in PER-acyp). Two genes were not annotated (AALB000689 (FC = 87.346 in PER-delta, FC = 314.417 in PER-acyp and AALB000331 (FC 36.980 in PER-delta, FC 41.413 in PER-acyp). Among the major detoxification enzyme families, the pattern of overexpression is broadly similar to that seen in deltamethrin resistant mosquitoes, with CYP6 cluster genes overexpressed, though the X chromosome genes CYP9K1 (AALB003283) and CYP6M1 (AALB015585) were less highly overexpressed in alpha-cypermethrin-resistant than in deltamethrin-resistant mosquitoes. Six putative UDP-glucuronosyltransferases were common in both PER-delta and PER-acyp at comparable levels (Table 2). Similarly, a group of cytochrome P450 genes were shared between the two samples with an exception of CYP9J5 (AALB010082), CYP307A1 (AALB006365), CYP4H15/CYP4H25/CYP4H27 (AALB015703) and CYP6Y2 (AALB015574) which were only DE in the PER-delta group while CYP4C26 (AALB015526), CYP4H18 (AALB001475) and two putative cytochrome P450 genes (AALB015526 and AALB010702) were only in the PER-acyp group (Table 2). Other genes related to insecticide detoxification that were common between deltamethrin and alpha-cypermethrin resistant genes were thirteen cuticular proteins with only one upregulated in both samples (AALB001993, PER-delta FC = 2.128 and FC = 2.222 PER-acyp) and one carboxylesterase that was also downregulated in both samples (AALB007549, FC = 0.18 in PER-delta and FC = 0.35 in PER-acyp).

### Validation of relative expression levels estimated by RNA-Seq with quantitative RT-PCR

Quantitative RT-PCR was used to validate the RNA-Seq results of seven genes among the most overexpressed. The qRT-PCR results broadly supported the directionality of the changes in expression levels, although for several genes, the magnitude of the expression difference was smaller than estimated by RNA-Seq (S1 Fig). The overexpression in the PER-acyp and PER-unx were very similar probably due to overall high levels of resistance in field populations. The overexpression of the CYP4C26 gene detected by qRT-PCR was different possibly due to RNA-Seq over-estimation.

**Fig 6:**
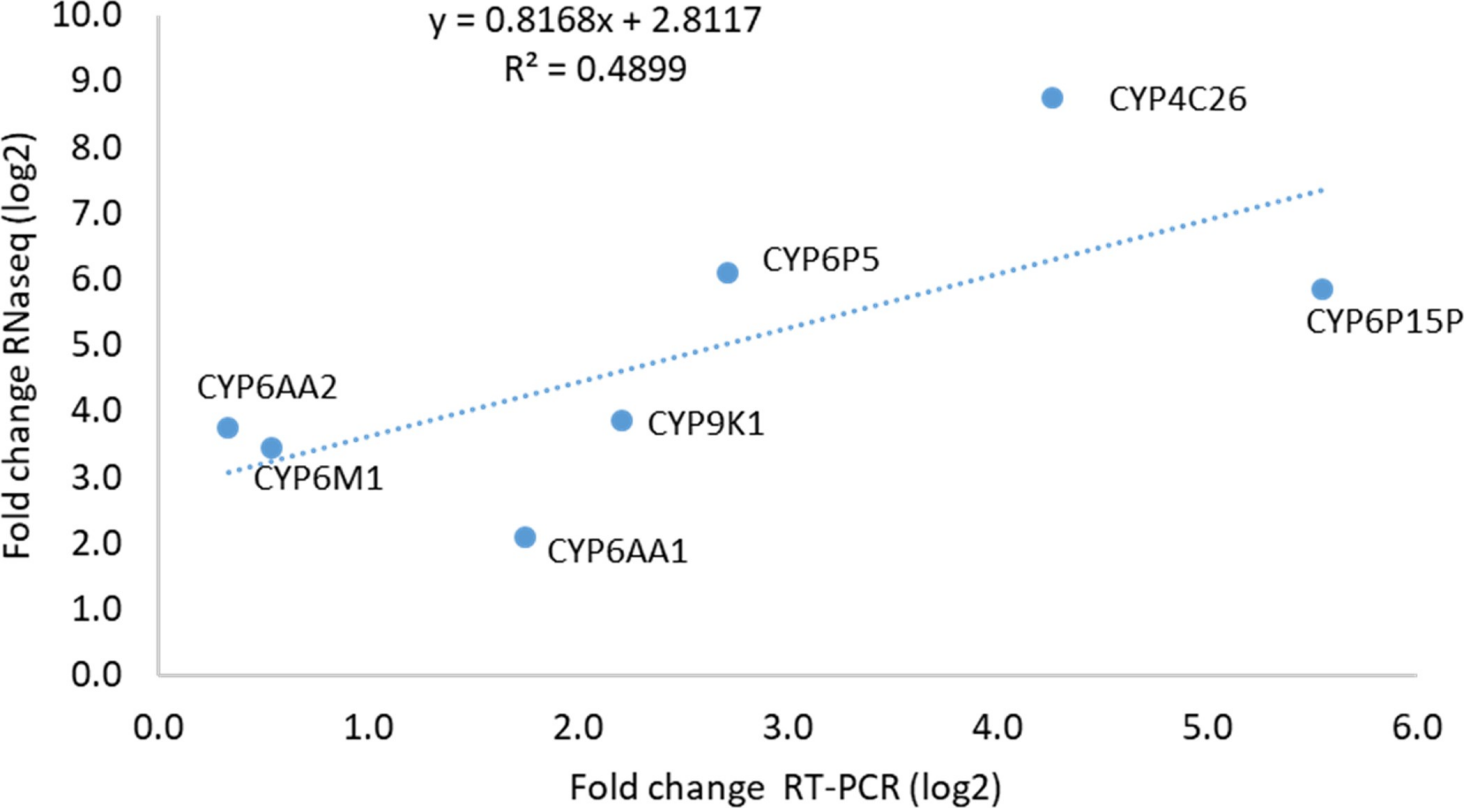
Correlation between RNA-Seq and qRT-PCR data of selected genes from the list of the up regulated transcripts.

A significant correlation between the qRT-PCR and RNA-seq results was observed as shown in Fig 6 below (R^2^ = 0.489; P = 0.004).

### Identification of target site mutations from RNA-Seq data

RNA-Seq alignments were used to identify and roughly quantify target site mutations associated with insecticide resistance (Tables 3, 4 and 5).

Target site mutations in the *para* voltage gated sodium channel (VGSC) gene (target site of pyrethroids and DDT) have previously been reported in *An*. *albimanus* [53]. Two resistance-associated amino acids at codon 1014, TCG (Serine) and TGT (Cysteine), were detected at low levels in some Peruvian samples but were not detected in any Guatemalan samples (Table 3). In unexposed control mosquitoes from Peru, TCG (Ser) was seen at 0%, 7% and 10% in the three replicate pools. In deltamethrin-resistant mosquitoes from Peru, no resistance alleles were detected, while in alpha-cypermethrin-resistant mosquitoes, one pool showed 0% resistant alleles, in another TCG (Ser) was seen at 14% and in another TGT (Cys) was seen at 26% (Table 3). While these counts indicate the presence of a codon and an approximate quantification, they cannot be directly translated into allele frequencies as the sequence reads are sampled from a pool of transcripts from a pool of mosquitoes, with potentially different levels of contribution from each mosquito and allele. In order to appropriately represent the allele frequency in the different populations, allele frequency should be determined from much larger numbers of individual mosquitoes. However, the pools used give a broad picture of point mutations segregating in each population.

**Table 3:** Codons present in RNA-seq reads at the *kdr* position ‘1014’ of the voltage gated sodium channel gene.

		Susceptible	Susceptible	Susceptible	Resistant	Resistant
Sample	Coverage	TTG (Leu)	CTG (Leu)	TTA (Leu)	TCG (Ser)	TGT (Cys)
STECLA	1	**1**	0	0	0	0
San-1	85	**85**	0	0	0	0
San-2	76	**76**	0	0	0	0
San-3	65	**64**	**1**	0	0	0
GTM-unx-1	61	**60**	0	**1**	0	0
GTM-unx-2	29	**29**	0	0	0	0
GTM-unx-3	31	**31**	0	0	0	0
GTM-delta-1	23	**23**	0	0	0	0
GTM-delta-2	10	**10**	0	0	0	0
GTM-delta-3	16	**16**	0	0	0	0
PER-unx-1	52	**52**	0	0	0	0
PER-unx-2	75	**70**	0	0	**5**	0
PER-unx-3	29	**26**	0	0	**3**	0
PER-delta-1	67	**67**	0	0	0	0
PER-delta-2	42	**41**	**1**	0	0	0
PER-delta-3	25	**25**	0	0	0	0
PER-acyp-1	29	**25**	0	0	**4**	0
PER-acyp-2	34	**34**	0	0	0	0
PER-acyp-3	19	**13**	**1**	0	0	**5**

**Table 4:** Codons present in RNAseq reads at position ‘119’ of the Acetylcholinesterase-1 gene.

		Susceptible	Resistant	Unknown
Sample	Coverage	GGC (Gly)	TCC (Ser)	GCC (Ala)
STECLA	1	**1**	0	0
San-1	6	**6**	0	0
San-2	3	**3**	0	0
San-3	3	**3**	0	0
GTM-unx-1	3	**3**	0	0
GTM-unx-2	2	**2**	0	0
GTM-unx-3	2	**2**	0	0
GTM-delta-1	3	**3**	0	0
GTM-delta-2	0	0	0	0
GTM-delta-3	3	**3**	0	0
PER-unx-1	5	0	**3**	**2**
PER-unx-2	5	0	**2**	**3**
PER-unx-3	11	**2**	**7**	**2**
PER-delta-1	15	0	**10**	**5**
PER-delta-2	12	**1**	**10**	**1**
PER-delta-3	6	**1**	**5**	0
PER-acyp-1	10	0	**6**	**4**
PER-acyp-2	17	**2**	**13**	**2**
PER-acyp-3	2	0	**2**	0

**Table 5:** Codons present in RNAseq reads at position ‘296’ of the GABA gated chloride channel A subunit gene.

		Susceptible	Resistant	Unknown	Unknown
Sample	Coverage	GCA (Ala)	TCA (Ser)	CCA (Pro)	GTA (Val)
STECLA	1	**1**	0	0	0
San-1	20	0	**20**	0	0
San-2	40	0	**38**	**2**	0
San-3	44	0	**44**	0	0
GTM-unx-1	12	**11**	**1**	0	0
GTM-unx-2	6	**4**	**2**	0	0
GTM-unx-3	10	**10**	0	0	0
GTM-delta-1	10	**9**	0	0	**1**
GTM-delta-2	17	**15**	**2**	0	0
GTM-delta-3	2	**2**	0	0	0
PER-unx-1	16	0	**16**	0	0
PER-unx-2	22	0	**22**	0	0
PER-unx-3	9	0	**9**	0	0
PER-delta-1	36	0	**36**	0	0
PER-delta-2	21	0	**21**	0	0
PER-delta-3	17	0	**17**	0	0
PER-acyp-1	5	0	**5**	0	0
PER-acyp-2	10	0	**10**	0	0
PER-acyp-3	6	0	**6**	0	0

The samples were also analysed to detect known resistance mutations associated with other, non-pyrethroid insecticides. Target site mutations in the Acetylcholinesterase-1 (ACE-1) gene (target of carbamates and organophosphates) have been reported in *An*. *albimanus* [54]. A marked contrast between the two study populations was seen, with a resistance allele predominating in Peruvian samples while completely absent from the Guatemalan samples (Table 4). This allele, TCC (Ser), was seen at 40%, 60%, 63.64% in unexposed mosquitoes, at 67%, 83% and 83% in deltamethrin-resistant and 60%, 76% and 100% in alpha-cypermethrin-resistant mosquitoes. Another allele, GCC (Ala), was common (up to 60%) in some of the Peruvian samples, while absent from Guatemalan samples, though whether this allele confers resistance is not known. No target site mutations in the GABA gated chloride channel A (GABA-a; target of the banned pesticide dieldrin, as well as ivermectin and fipronil) have been reported in *An*. *albimanus*. Again, differences were detected between the two populations, with a resistance allele, TCA (Ser), apparently fixed in the Peruvian population (100% frequency in all samples) and seen at lower frequencies in the Guatemalan samples (between 0% and 33%). The susceptible Sanarate strain also appears to be nearly fixed for TCA (Ser), with 95%, 100% and 100% frequencies in the three replicate pools. Two alleles of unknown resistance status, CCA (Pro) and GTA (Val), were detected at low levels in two Sanarate samples (Table 5).

### Identifying Cytochrome Oxidase I (COI) haplogroups of field-collected *An*. *albimanus* from Guatemala and Peru

To identify genetic populations to which the field-collected mosquitoes belonged, we identified the genotypes of the COI and compared them to published data for this gene (S2 Fig). The Peruvian population sequences clustered with those from Pacific Colombia, while both the Sanarate and Guatemalan sequences clustered with a subset of sequences from Panama, which appears to be a heterogeneous population, possibly reflecting its location linking Central America and South America. Understanding the population structure of *An*. *albimanus* is important for predicting how insecticide resistance might spread across the species’ range and the genetic background against which it arises. The results show that the populations from Guatemala and Peru belong to different haplogroups and that barriers to gene flow in addition to geographical distance may exist that could limit the spread of particular resistance mechanisms among mosquito populations.

## Discussion

Unlike in sub-Saharan Africa where multiple research groups are actively elucidating the complex biochemistries and molecular bases for insecticide resistance in malaria vectors, very little is known about the mechanisms that underpin insecticide resistance in malaria vectors in the Americas. The results from this study demonstrated that *An*. *albimanus* from the study locations in Peru and Guatemala were resistant to the two pyrethroids tested, deltamethrin and alpha-cypermethrin and this resistance is mainly metabolic driven by over-expression of cytochrome P450s.

In the samples from Peru, several detoxification genes from the cytochrome P450 monooxygenase family that have previously been associated with pyrethroid resistance in other species were highly overexpressed. These include, CYP6P5, CYP9K1, CYP6AA2, CYP6Z2/CYP6Z3, CYP6AA1, CYP6P15P, CYP6M1, CYP6M4, CYP6P3, CYP6P4 and CYP6M3. There were no significant differences in gene expression levels when comparing the deltamethrin and alpha-cypermethrin samples at p<0.01. However, a single P450 gene CYP4C26 was overexpressed 9.8 fold at p<0.05 in the alpha-cypermethrin resistant samples. Functional validation of the role of this gene in alpha-cypermethrin metabolism could provide a candidate gene that could potentially be used to distinguish resistance between the two pyrethroids. Similarity of gene expression for mosquitoes resistant to both pyrethroids suggests that genes involved are likely metabolically efficient to detoxify most pyrethroids as seen in other mosquitoes species for genes such as CYP6P3 and CYP6M2 in *An*. *gambiae* [55, 56] or CYP6P9a/b and CYP6M7 for *An*. *funestus* [19, 57].

Among the genes found to be overexpressed in the resistant mosquitoes, CYP6AA2 has been previously reported to be associated with deltamethrin resistance in *Anopheles minimus* [58]. CYP6AA1 has also been reported to be overexpressed in *An*. *gambiae* from Burkina Faso resistant to deltamethrin and permethrin [59] and was recently shown to be able to metabolise both type I and type II pyrethroids in *An*. *funestus* [60]. This suggests that some resistance mechanisms may be shared across *Anopheles* species worldwide probably as a result from shared ancestral evolutionary adaptation to xenobiotics.

Genes among the top 10 commonly shared in GTM-delta, PER-delta and PER-acyp when the resistant versus susceptible and control versus susceptible groups were compared were genes belonging to serine-type endopeptidase and genes related to extracellular space in GO terms. Serine-type endopeptidases were also reported to be enriched in population resistant to permethrin and deltamethrin [61] and also in DDT resistant strains of *An*. *gambiae* [62]. Serine type proteases involved in immune responses have been isolated in *An*.*gambiae* [63] hence the up-regulation of these proteases may act as defence mechanisms against the exposure to insecticide.

The most striking results however, were from the comparison of the deltamethrin resistant samples from the two locations in Peru and Guatemala with the susceptible laboratory strain. There was a marked contrast in gene expression specifically from the cytochrome P450 monooxygenase family. When taking in to consideration only the genes that were commonly expressed in the comparison of resistant versus susceptible and control versus susceptible, in deltamethrin-resistant samples from Guatemala, there were 28 differentially expressed cytochrome P450s with a fold change ranging from 5.0 to 0.12. Of these, only two were significantly overexpressed, CYP6M1 with a fold change of 5.0 at p<0.01 and CYP4235 with fold change of 2.08 at p<0.05. It is not clear why many of the differentially expressed genes related to insecticide detoxification were down-regulated in the Guatemala samples; this may have been due to an overall lower frequency of pyrethroid resistance in this population, as evidenced by the bioassay data. On the other hand, the deltamethrin resistant samples from Peru had 17 differentially expressed cytochrome P450s with a fold change ranging from 68.60 to 0.23. Of these, 15 significantly upregulated at p<0.01 with a fold change ranging from 68.60 to 2.48. The main cytochrome P450s overexpressed more than 10 times were CYP6P5 FC = 68.6, CYP6P15P FC = 58.1, CYPAA2 FC = 26.75, CYP9K1 FC = 14.43 and CYP6M1 FC = 10.93.

Two overexpressed insecticide detoxification genes that were shared between the two groups using the resistant susceptible pairwise comparison were CYP026 (AALB015585) and a putative UDP-glucuronosyltransferase (AALB000333). Functional validation of these genes may indicate that they play an important role in deltamethrin detoxification regardless of the geographical location.

The striking difference in transcriptional profiles of mosquitoes from the two locations could indicate a systematic difference between the samples. A major limitation of this study was our inability to rear offspring of field collected mosquitoes and thereby control for the age of the adult mosquitoes tested in the bioassays. A systematic difference in the age profile of the mosquitoes tested could affect the gene expression profiles seen. Indeed, the Guatemalan mosquitoes showed over-expression of many genes associated with DNA replication and cell cycle processes, perhaps indicating young, developing mosquitoes with actively proliferating tissues. Age is negatively correlated with ability to survive insecticide exposure, which may account for the survival of a subset of the exposed mosquitoes. However, using wild caught samples allows for the opportunity to fully mimic adaptive processes occurring in nature. Comparison of these samples to the fully susceptible laboratory strain enabled the detection of differences arising due to factors unique to their respective natural environments. The results for the Peruvian samples clearly show a transcriptomic profile that unmistakably implicates a group of known resistance genes.

In addition, the differences in gene expression profiles could at least partially arise from other types of selection pressures unique to the two geographical settings studied. In this study, samples from Guatemala were collected in close proximity to sugarcane, palm oil and banana plantations while the samples from Peru were collected in close proximity to rice and banana fields. Previous studies have reported the influence of agriculture on the evolution of insecticide resistance in mosquitoes [64–66]. Regional variations in agricultural pesticide use may have contributed to the selection pressures on the two populations studied here, although further research would be needed to determine the predominant agrochemicals and their frequency of application around mosquito habitats.

There was no significant difference in gene expression between the resistant samples and the unexposed samples. This is perhaps due to the fact that resistance is mainly conferred through a constitutive over-expression of resistance genes in the respective samples, i.e. PER-unx, PER-delta, PER-acyp, and GTM-unx and GTM-delta. The difference in over-expression of the detoxification genes was not high enough to be captured by the differential gene expression analysis.

Most of the current DNA markers available for monitoring resistance to pyrethroids are based on the detection of mutations on insecticide target sites. Multiple studies are currently underway in an effort to identify markers of metabolic resistance, as these mechanisms are now thought to be the primary cause of vector control failure. The use of high-throughput sequencing techniques such as RNA sequencing utilized in this study, provide an unprecedented level of detail which in turn led to the identification of enormous numbers of differentially expressed genes. This, however, presents a challenge in the identification of candidate markers underlying resistance. In addition, most metabolic detoxification genes belong to large gene families and are seen to be species specific. In *An*. *gambiae*, CYP6P3 and CYP6M2 have been shown to metabolize permethrin and deltamethrin [20, 56, 67]. While in the Asian malaria vector *An*. *minimus* two P450s, CYP6P7 and CYP6AA3, have been shown to metabolize the pyrethroids permethrin, cypermethrin and deltamethrin [68, 69]. As detected in the present study, geography can also play a role in mechanism heterogeneity such as noted in the significant differences in the resistance profiles of *Anopheles funestus* from Zambia, Mozambique and Malawi [19]. As a consequence, novel markers specific to species and also to geographical region may be required. However, with the cost of sequencing steadily decreasing, identification of these markers will become more achievable and help to provide a more robust basis for understanding and managing insecticide resistance.

In conclusion, *An*. *albimanus* mosquitoes from Guatemala in Central America and from the Pacific coast of Peru in South America showed contrasting patterns reflected in putatively different resistance mechanisms. Both showed resistance to the pyrethroids deltamethrin and alpha-cypermethrin, but the Peruvian population appeared to be more highly resistant to alpha-cypermethrin than the Guatemalan population. Transcriptome profiling showed contrasting patterns of gene expression between the two populations. In the Peruvian population, a number of detoxification genes that have been implicated in metabolic resistance to insecticides in other mosquito species were found to be highly overexpressed. Target-site mutations associated with resistance to pyrethroids and other insecticides also appeared to be more common in the Peruvian mosquitoes. The study identifies genes associated with pyrethroid resistance in *An*. *albimanus* but highlights the challenges related to geographic heterogeneities in resistance mechanisms.

## Supporting information

S1 Table Descriptive statistics of RNA-Seq reads and alignments. Table of key statistics describing RNA-Seq datasets before and after processing and alignment to the reference genome.
(XLSX)

S2 Table Functional annotation of AalbS2.1 protein coding genes. Full results of blast2go analysis to assign functional annotation and gene ontologies to predicted proteins from the *Anopheles albimanus* reference gene set AalbS2.1.
(XLSX)

S3 Table Oligonucleotide primers used for qRT-PCR. Oligonucleotide primer pairs, expected amplicon sizes and primer efficiencies, calculated from standard curves, for genes analysed in this study.
(XLSX)

S4 Table Results of differential gene expression analyses. Full results of pairwise differential gene expression analyses of *Anopheles albimanus* RNA-Seq datasets.
(XLSX)

S5 Table Gene ontology enrichment analysis results. Full results of gene ontology enrichment analysis for differentially expressed gene sets.
(XLSX)

S6 Table Descriptive statistics of RNA-Seq alignments to the cytochrome oxidase I (COI) gene. Table of key statistics describing RNA-Seq alignments to a representative COI gene sequence.
(XLSX)

S1 Fig Relative expression levels estimated by RNA-Seq and qRT-PCR. Relative gene expression levels among Peruvian mosquitoes exposed to alpha-cypermethrin, unexposed mosquitoes and unexposed mosquitoes from the Sanarate colony. The y-axis shows log2 fold-change for each pairwise comparison. Error bars are not shown for the RNAseq log2 fold-change estimates from edgeR analysis (3 biological replicates for each condition) as they are not informative for these estimates. For the qPCR data, log2 fold-change estimates were the negative deltdeltaCt values. Three biological replicates for each condition and each gene, each with 3 technical replicates, were used to calculate the mean deltaCt (of the 3 biological replicates) and its standard error. The SEM of the negative deltdeltaCt values (i.e. the log2 fold-change estimates) was calculated using Gauss' error propagation (the square root of the sum of squared SEM for each condition compared) and +/- 1 SEM was shown for each bar. Significant differential expression (tested by edgeR for the RNAseq data and by a two sample t-test of deltaCt values for qPCR) is indicated above each bar (1 asterisk indicates p<0.05; 2 asterisks indicate p<0.01; 3 asterisks indicate p<0.001).
(TIF)

S2 Fig Multidimensional Scaling analysis plot of Cytochrome Oxidase I (COI) of *An*. *albimanus*. Haplogroups of field collected *An*. *albimanus* from Guatemala and Peru based on Cytochrome Oxidase I. Samples are as labelled on the legend.
(PDF)
