# Supplementary figures and images for: Contrasting patterns of gene expression indicate differing pyrethroid resistance mechanisms across the range of the New World malaria vector Anopheles albimanus
Source: PLoS One. 2019 Jan 30;14(1):e0210586. doi: 10.1371/journal.pone.0210586 (PMC6353143; doi:10.1371/journal.pone.0210586)

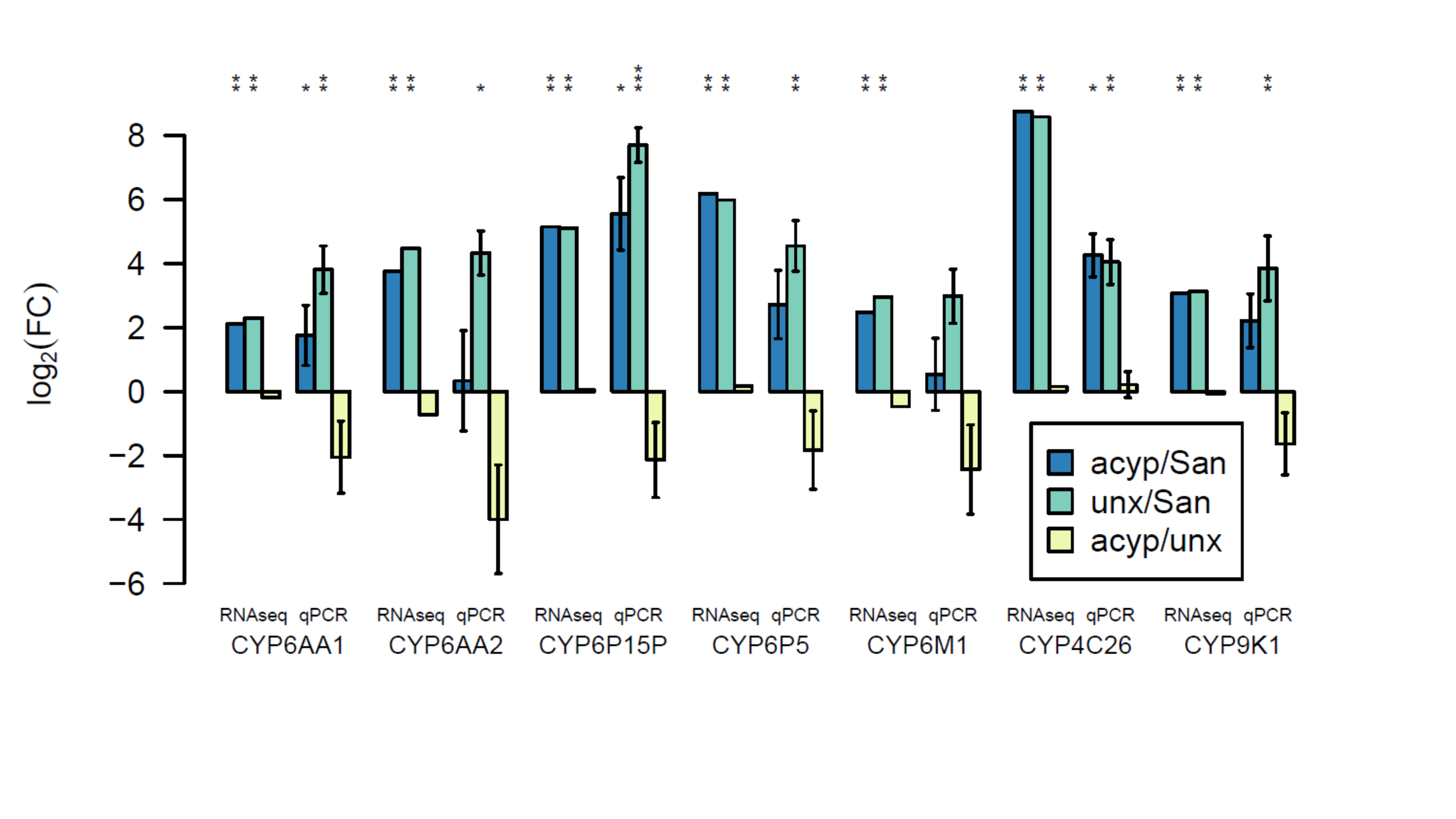

Supplement: S1 Fig — Relative gene expression levels among Peruvian mosquitoes exposed to alpha-cypermethrin, unexposed mosquitoes and unexposed mosquitoes from the Sanarate colony. The y-axis shows log2 fold-change for each pairwise comparison. Error bars are not shown for the RNAseq log2 fold-change estimates from edgeR analysis (3 biological replicates for each condition) as they are not informative for these estimates. For the qPCR data, log2 fold-change estimates were the negative deltdeltaCt values. Three biological replicates for each condition and each gene, each with 3 technical replicates, were used to calculate the mean deltaCt (of the 3 biological replicates) and its standard error. The SEM of the negative deltdeltaCt values (i.e. the log2 fold-change estimates) was calculated using Gauss' error propagation (the square root of the sum of squared SEM for each condition compared) and +/- 1 SEM was shown for each bar. Significant differential expression (tested by edgeR for the RNAseq data and by a two sample t-test of deltaCt values for qPCR) is indicated above each bar (1 asterisk indicates p<0.05; 2 asterisks indicate p<0.01; 3 asterisks indicate p<0.001). (TIF) [file pone.0210586.s007.tif]

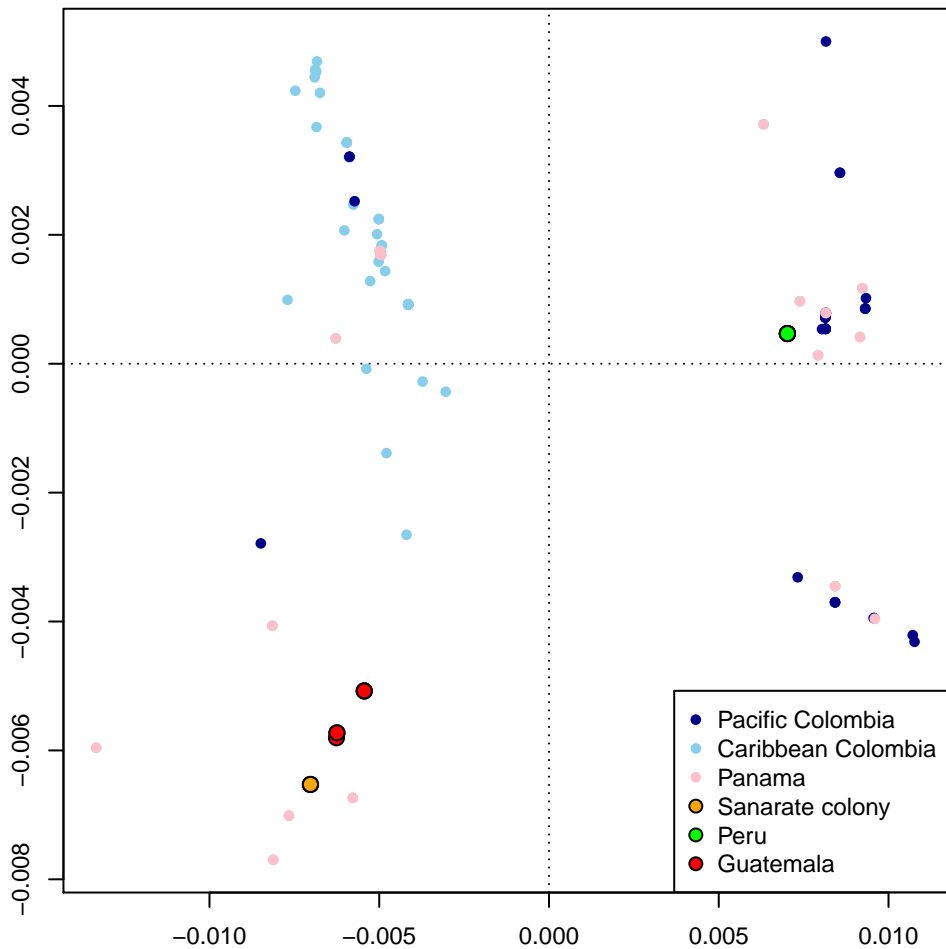

Supplement: S2 Fig — Haplogroups of field collected An. albimanus from Guatemala and Peru based on Cytochrome Oxidase I. Samples are as labelled on the legend. (PDF) [file pone.0210586.s008.pdf]
